# Supplementary material for: The impact of social support on career decision-making difficulties: The serial mediating roles of career decision-making self-efficacy and job search clarity, and moderation by proactive personality
Source: PLoS One. 2026 Mar 9;21(3):e0344515. doi: 10.1371/journal.pone.0344515 (PMC12970892; doi:10.1371/journal.pone.0344515)
Supplement: Attachment — Submitted filename: Response to Reviewers.docx [file pone.0344515.s004.docx]

Reviewer #1: Abstract

1. The abstract does not include key statistical results (e.g., effect sizes, p-values).

It uses technical terms (e.g., “moderated mediation”) without brief clarification for general readers.

For improvement:

Include a sentence summarizing key statistical findings. Clarify how constructs (e.g., job search clarity, self-efficacy) were measured. Consider streamlining wording for clarity and flow.

Response: Thank you very much for your constructive comments on our manuscript. We have carefully considered your suggestions and have revised the abstract accordingly. Below is a detailed response to your specific points:

(1) We have now included a sentence summarizing the key statistical findings in the abstract. (2) We have clarified the constructs and measurement tools used in the study. For example, we have briefly explained the concept of "moderated mediation" in the context of our findings. (3) We have revised the wording to improve clarity and readability.

The revised section of our manuscript now reads:

“Based on the Social Cognitive Model of Career Self-Management, this study explored how social support was related to career decision-making difficulties among 991 vocational college students. Social support was measured through the Perceived Social Support Scale, career decision-making self-efficacy with the Career Decision-Making Self-Efficacy Questionnaire, job search clarity via the Job Search Clarity Questionnaire, career decision-making difficulties using the Career Decision-Making Difficulties Questionnaire, and proactive personality by the Proactive Personality Scale. The results showed that: Job search clarity partially mediate the relationship between social support and career decision-making difficulties. The indirect effect was significant, β = -0.08, p < 0.001, accounted for 20% of the total effect. In addition, career decision-making self-efficacy and job search clarity jointly serve as serial mediators in the link between social support and career decision-making difficulties. The serial indirect effect was significant, β = -0.12, p < 0.001, accounting for 30% of the total effect. Furthermore, proactive personality moderates the mediating role of job search clarity, particularly in the second stage of the mediation. Specifically, the effect of job search clarity on career decision-making difficulties became more pronounced as proactive personality increased (β = -0.04, p < 0.001). These findings extend the social cognitive model of career self-management by demonstrating the sequential mediating roles of career decision-making self-efficacy and job search clarity, and the moderating role of proactive personality, in the link between social support and reduced career decision-making difficulties. Practically, they highlight the importance of designing career interventions for vocational college students that simultaneously target social support networks, self-efficacy enhancement, and clarity-building activities, particularly for students lower in proactive personality.”

2. Introduction

Clearly identifies the gap in understanding how social support influences career decision-making.

For improvement:

The transition from literature to hypotheses is occasionally abrupt and could benefit from clearer signposting.

Response: We appreciate your insightful comment regarding the transition from literature to hypotheses. We fully agree that a smoother and more logical transition is essential for the clarity and coherence of the introduction. To address this issue, we have made the following revisions: (1) We have added transitional sentences and paragraphs to bridge the gap between the literature review and the hypotheses. (2) We have used more explicit signposting words and phrases to guide the reader through the text.

The revised section of our manuscript now reads:

Based on the existing literature, it is evident that social support plays a significant role in influencing CDMD. However, the specific mechanisms and potential moderating factors involved in this relationship remain unclear. Therefore, we firstly hypothesize that social support will act as a mitigating factor, reducing the degree of CDMD among vocational college students.

Hypothesis 1: …

Considering these inconsistent findings, this study aims to further explore the mediating role of CDSE in the relationship between social support and CDMD among vocational college students. Accordingly, the hypothesis is formulated as follows (Mediating Effects):

Hypothesis 2a: …

To further explore the mechanisms underlying CDMD, this study examines the mediating role of job search clarity in the relationship between social support and CDMD. As such, the following hypotheses are proposed (Mediating Effects):

Hypothesis 2b: …

Proactive personality, closely related to openness, conscientiousness, extraversion, and agreeableness in the Big Five personality traits [37], is seen as an extension and deepening of the meanings of the Big Five personality model [38]. Given the importance of proactive personality in influencing adaptive career behaviors, this study aims to explore its moderating role in the relationship between CDSE, job-search clarity, and CDMD among vocational college students. Thus, the study puts forward the following hypotheses (Moderating Effects):

Hypothesis 3a: …

Hypothesis 3b: …

3. Literature Review and Theoretical Foundation

A more critical discussion of conflicting findings (e.g., regarding mediation by self-efficacy) would strengthen the justification for the study.

Redundancy appears in some paragraphs (e.g., repeated phrasing of concepts like “career decision-making difficulties”).

For improvement:

Streamline and synthesize key findings from the literature to improve flow and reduce repetition.

Response: Thank you very much for your insightful comments on my manuscript. We have carefully considered your suggestions regarding the literature review and theoretical foundation, and have made the following revisions to address your concerns: (1) We have added a detailed analysis of the inconsistencies in the research findings regarding the mediating role of career decision-making self-efficacy. (2) Thank you for pointing out the redundancy in my manuscript. We have eliminated redundant phrasing and focused on key information.

The revised section of our manuscript now reads:

(1) These inconsistent findings may originate from variations in sample characteristics, measurement tools, and research designs. For instance, Jemini-Gashi et al.'s study focused on a specific population of students. In contrast, the Taiwan study employed different measures for CDSE and CDMD.

4. Hypotheses and Research Objectives

for Improvement:

The hypotheses could be numbered or visually separated for easier readability.

The “serial mediation” and “moderated mediation” could be better defined for readers unfamiliar with these concepts.

Response: We have revised the presentation of the hypotheses to improve readability. Each hypothesis is now clearly numbered and separated visually, making it easier for readers to follow and reference them. To ensure clarity for readers who may not be familiar with these concepts, we have added definitions and brief explanations of "serial mediation" and "moderated mediation" in the introduction. This will help readers understand the specific mechanisms being tested in the study.

5. Language, Structure, and Clarity

Minor language editing is needed for fluency and consistency.

Standardize formatting of terms like “job-search clarity” and “career decision-making.”

The hypotheses could be presented more clearly. While the general aim is stated, specific hypotheses related to each mediating and moderating effect are not always well-articulated.

Response: We have conducted a thorough language edit to improve fluency and consistency throughout the manuscript. Additionally, We have standardized the formatting of key terms such as "job-search clarity" and "career decision-making" to ensure uniformity.

6. Further discussion on sample diversity (e.g., gender, socioeconomic status) could provide more insight into whether the findings are influenced by these factors.

Response: Thank you for the constructive feedback on our manuscript. We recognize the importance of sample diversity and appreciate the suggestion to further discuss its potential impact. However, after careful consideration, we believe that incorporating an in-depth exploration of gender and socioeconomic status may deviate from the primary focus of this study. Our research centers on the relationship between social support and career decision-making difficulties among vocational college students, with an emphasis on the mediating and moderating factors within this specific context.

7. Statistical Analysis and Assumptions:

While the statistical methods appear sound, the assumptions behind the mediation and moderation analysis could be discussed in more detail (e.g., assumptions of normality, homogeneity of variance).

The paper would benefit from a goodness-of-fit test for the structural model to provide confidence in the proposed relationships.

Response: We are grateful for the insightful feedback on our manuscript. The suggestions to elaborate on the assumptions behind the mediation and moderation analysis, as well as to include a goodness-of-fit test for the structural model, have been carefully considered and addressed.

The revised section of our manuscript now reads:

(1) Before conducting the mediation analysis, we assessed the assumptions underlying the mediation model. The assumptions of normality and homogeneity of variance were examined. The Shapiro-Wilk test indicated that the residuals of all variables were approximately normally distributed (p > 0.05). Levene's test was used to check the homogeneity of variance, and no significant differences were found in the variances of the outcome variable across different levels of the predictor variables (p > 0.05), suggesting that the assumptions for mediation analysis were met.
(2) Before estimating the models, the assumptions for the moderation analysis were assessed. The interaction terms were examined for multicollinearity, and the variance inflation factors (VIFs) were all below 10, indicating that multicollinearity was not a significant issue. For this analysis, two models were estimated. Model 0 included the main effect of proactive personality in the mediation model.

8. Discussion and Limitations:

The discussion of results is somewhat limited. The authors could better integrate the findings into the broader literature, noting contradictions, confirmations, and unexplored areas.

Limitations are briefly mentioned but not thoroughly addressed. A more detailed discussion on the limitations, particularly concerning the cross-sectional design, would be helpful.

Recommendations for Improvement:

Refinement of Hypotheses:

Clearly define specific hypotheses and how each hypothesis is tested in the analysis. This would improve the structure and readability of the results section.

Broader Implications:

While the practical implications are noted, these can be expanded. The authors can suggest actionable strategies for educators, career counselors, and policymakers, particularly in vocational settings.

Future Research Directions:

The paper could provide more suggestions for future research, particularly related to longitudinal studies to examine causality, and expanding the sample to include other educational institutions or regions.

Discussion of Cultural Considerations:

Given the focus on vocational students, there may be cultural influences on career decision-making that can be explored in greater detail. Cultural factors that influence career choice in vocational students could be discussed, especially since the study is based on Chinese students.

Response: Thank you for your feedback. We have carefully revised the discussion section to better integrate the findings into the broader literature and address the limitations more thoroughly. We've revised the limitations section to provide a more detailed discussion, particularly regarding the cross-sectional design.

The revised section of our manuscript now reads:

Limitations and Future Research Directions

Notwithstanding its substantive contributions to understanding career decision-making difficulties among vocational college students, this investigation is circumscribed by several methodological and conceptual limitations that warrant deliberate attention in subsequent scholarship. Particular exigencies surrounding the cross-sectional design merit prioritized resolution to advance the epistemological foundations of this domain.

Primarily, the exclusive dependence on monomethod self-report measures introduces non-trivial risks of common method variance—a psychometric artifact potentially confounding observed relationships. Future research should circumvent this constraint through triangulated assessment protocols, incorporating multi-informant data (e.g., parental, pedagogical, or employer perspectives) to cultivate richer ecological validity and attenuate measurement artifact contamination. Complementarily, implementing temporal separation of predictor-criterion measurements across distinct assessment waves would further dismantle method covariance while fortifying inferential robustness.

Secondly, the inherent constraints of cross-sectional topology fundamentally preclude causal attribution among examined constructs. While statistically significant associations emerged linking social support, career decision-making self-efficacy, job search clarity, proactive personality, and career decision-making difficulties, these relational patterns demand circumspect interpretation absent temporal precedence evidence. Prospective studies must adopt longitudinal or experience-sampling methodologies to elucidate developmental cascades and reciprocal dynamics. Sequential data capture would unravel causal sequences and bidirectional influences, ultimately mapping ontogenetic trajectories of these psychosocial phenomena.

Thirdly, the deliberate sampling frame—restricted to Chinese vocational college cohorts—simultaneously enables contextual depth yet constrains population generalizability. To transcend these geographical and institutional boundaries, future inquiries should incorporate comparative samples spanning diverse educational echelons (e.g., undergraduate, postgraduate) and cross-national vocational training ecosystems. Such strategic expansion would critically interrogate the external validity frontier while delineating universal versus context-bound manifestations of career decision-making difficulties.

Fourthly, the counterintuitive null finding regarding career decision-making self-efficacy 's direct predictive utility contravenes established theoretical postulates, signaling latent mechanistic complexity. This empirical anomaly necessitates rigorous deconstruction of mediating pathways—potentially involving vocational identity crystallization or goal-regulation architectures—that may obfuscate the career decision-making self-efficacy - career decision-making difficulties nexus. Elucidating these covert regulatory processes promises to recalibrate theoretical models and resolve extant theoretical discordances.

Finally, the conspicuous omission of cultural-psychological determinants represents a critical lacuna. Within China's collectivist milieu, familial expectations, sociostructural prescriptions, and gendered occupational norms profoundly scaffold developmental trajectories of career volition. Future research must therefore: (a) systematically integrate cultural moderators (e.g., filial piety, gender role ideology) to map their contingent interactions with psychological antecedents of career decision-making difficulties; and (b) launch coordinated cross-cultural programmes contrasting individualist versus collectivist vocational ecosystems. Such initiatives would disentangle cultural universals from idiosyncrasies, forging culturally-attuned theoretical frameworks.

Reviewer #2: Thank you for the opportunity to review the manuscript. After careful evaluation, I am pleased to recommend the article for acceptance with minor revisions. The overall content is valuable and contributes meaningfully to the field; however, the title requires clarification to better reflect the scope and focus of the study. Additionally, the keywords should be reordered alphabetically. Once these minor adjustments are made, the manuscript will be suitable for publication.

Response: Thank you very much for your positive feedback and valuable suggestions. We have made the following adjustments to address your specific comments:

(1) We have revised the title to better reflect the scope and focus of the study. The new title is: "Social Support and Career Decision-Making Difficulties among Vocational College Students: The Roles of Mediation and Moderation" This revised title more clearly indicates the key variables and the analytical framework of our research, making it easier for readers to understand the main focus of our study. (2) We have reordered the keywords alphabetically to enhance the format consistency and ease of retrieval. The revised keywords are: "career decision-making difficulty; career decision-making self-efficacy; job search clarity; proactive personality; social support"
