# Supplementary material for: The impact of social support on career decision-making difficulties: The serial mediating roles of career decision-making self-efficacy and job search clarity, and moderation by proactive personality
Source: PLoS One. 2026 Mar 9;21(3):e0344515. doi: 10.1371/journal.pone.0344515 (PMC12970892; doi:10.1371/journal.pone.0344515)
Supplement: Attachment — Submitted filename: Associate Professor, Marwa Samir ,.docx [file pone.0344515.s002.docx]

**The impact of Social Support on Career Decision-1 Making Difficulties: The Serial Mediating Role of Career Decision-making Self-efficacy and Job Search Clarity and Moderation by Proactive Personality**

1. All modifications for all manuscript are done perfectly,
2. Abstract is clear,
3. Introduction: It is on the point
4. Experiments, statistics, and other analyses are performed to a high technical standard and are described in sufficient detail.
5. Conclusions are presented appropriately and are supported by the data.
6. The article is presented in an intelligible fashion and is written in standard English.
7. The research meets all applicable standards for the ethics of experimentation and research integrity.
8. The article adheres to appropriate reporting guidelines and community standards for data availability.
