# Supplementary material for: The impact of social support on career decision-making difficulties: The serial mediating roles of career decision-making self-efficacy and job search clarity, and moderation by proactive personality
Source: PLoS One. 2026 Mar 9;21(3):e0344515. doi: 10.1371/journal.pone.0344515 (PMC12970892; doi:10.1371/journal.pone.0344515)
Supplement: Attachment — Submitted filename: Reviewer Report PLOS One.docx [file pone.0344515.s003.docx]

**PLOS ONE**

Manuscript Number: PONE-D-25-12035R1

**Reviewer report: Associate Prof. Yasmeen Mohamed Mohamed Shehata**

**Point**

**Title****: The title clearly indicates the impact of Social Support on Career Decision- Making Difficulties: The Serial Mediating Role of Career Decision-making Self-efficacy and Job Search Clarity and Moderation by Proactive Personality

****Abstract**:**
- **Background**: limited context on the significance of the study

- **Objective**: Clearly states the aim of the study, which is to assesses explored how

11 social support was related to career decision-making difficulties among 991 vocational college students.

- **Methods**: Describes the number of participants, however neglects the study’s design
- **Results**: Summarizes key findings

- **Conclusion**: Highlights the impact of Social Support on Career Decision- Making Difficulties: The Serial Mediating Role of Career Decision-making Self-efficacy and Job Search Clarity and Moderation by Proactive Personality.

****Introduction****
- **Background/Rationale**:
- Discusses **Social Cognitive Model of Career Self-Management , Social Support and Career Decision-Making Difficulties , The Mediating Role of Career Decision-Making Self-Efficacy, The Mediating Role of Job Search Clarity, The Moderating Effect of Proactive Personality.**
- **Objectives**:
- Clearly states the specific aims and the study hypotheses, but it is preferred to list all hypotheses one after the other not in between the text.

. ****Methods****
- **Study Design**:
- Utilizes a cross-sectional survey was conducted, which is appropriate for examining variables.
- **Setting and Participants**:
- The study is conducted at a general hospital, with a clear description of the participant selection process.
- Sample size determination is not justified, moreover, what about the selection process and technique of participants.
- **Data Collection**:
- need more details about the year of development of each tool and revised versions

****Results****
- **Descriptive Statistics**:
- Presents demographic data of participants, including age, gender.
- **Correlation Analysis**:
- Provides clear statistical findings, highlighting significant correlations study variables

****Discussion****
- **Key Findings**:
- Summarizes the main results in relation to the study objectives and hypotheses

- **Comparison with Existing Literature**:
- Compares findings with previous studies, noting both consistencies and novel contributions to the field.

****Conclusion****
- Reiterates the importance of enhancing social support, fostering job search clarity, and strengthening proactive personality traits to alleviate CDMD among vocational college students

****Strengths and Limitations****
- neglect this section to be considered

**. **References****
- The manuscript includes a comprehensive list of references, demonstrating engagement with current literature and foundational theories relevant to the study.

**Summary**
The manuscript is well-structured and adheres to scientific research standards. It effectively addresses a pertinent issue among vocational college students

**To be added in limitation**

But there are some mistake according scientific research steps: -
. **Data Collection**
- **Reliance on Self-Reports**: Using subjective measures may lead to bias in responses
3. **Sample**
- **Sample Diversity**: If the sample consists only of vocational college students

, the results may not accurately reflect the experiences of all students across different vocational college
