# Supplementary material for: The impact of social support on career decision-making difficulties: The serial mediating roles of career decision-making self-efficacy and job search clarity, and moderation by proactive personality
Source: PLoS One. 2026 Mar 9;21(3):e0344515. doi: 10.1371/journal.pone.0344515 (PMC12970892; doi:10.1371/journal.pone.0344515)
Supplement: Attachment — Submitted filename: Response_to_Reviewers_auresp_2.docx [file pone.0344515.s005.docx]

**Reviewer #3:**

Reviewer’s comment:

*1. All modifications for all manuscript are done perfectly,*

*2.Abstract is clear,*

*3.Introduction: It is on the point*

*4.Experiments, statistics, and other analyses are performed to a high technical standard and are described in sufficient detail.*

*5.Conclusions are presented appropriately and are supported by the data.*

*6.The article is presented in an intelligible fashion and is written in standard English.*

*7.The research meets all applicable standards for the ethics of experimentation and research integrity.*

*8.The article adheres to appropriate reporting guidelines and community standards for data availability.*

**Author Response:** We are sincerely grateful to Reviewer for the thorough evaluation and the highly positive comments regarding our revised manuscript. We truly appreciate your recognition of the clarity of the abstract and introduction, the rigor of our statistical analyses, and the appropriateness of our conclusions. Thank you for your constructive review and supportive remarks, which have been highly motivating and helpful in refining our work.

**Reviewer #4:**

1. Reviewer’s comment: **Title**

*The title clearly indicates the impact of Social Support on Career Decision- Making Difficulties: The Serial Mediating Role of Career Decision-making Self-efficacy and Job Search Clarity and Moderation by Proactive Personality.*

**Author Response:** Thank you very much for your positive comment on the clarity and relevance of our title. After careful consideration, we made slight grammatical and stylistic refinements to further enhance precision and parallel structure. Specifically:

1.We changed “Role” to “Roles” because there are two mediating variables—career decision-making self-efficacy and job search clarity.

2.We added a comma after “Job Search Clarity”, and revised “and Moderation by Proactive Personality” to “and the Moderating Role of Proactive Personality” to maintain syntactic symmetry and consistency with the phrase “the Serial Mediating Roles.”

**Revised Title:** *The Impact of Social Support on Career Decision-Making Difficulties: The Serial Mediating Roles of Career Decision-Making Self-Efficacy and Job Search Clarity, and the Moderating Role of Proactive Personality*.

2. Reviewer’s comment: **Abstract**

*- **Background**: limited context on the significance of the study*

*- **Objective**: Clearly states the aim of the study, which is to assesses explored how social support was related to career decision-making difficulties among 991 vocational college students.*

*- **Methods**: Describes the number of participants, however neglects the study’s design*

*- **Results**: Summarizes key findings*

*- **Conclusion**: Highlights the impact of Social Support on Career Decision- Making Difficulties: The Serial Mediating Role of Career Decision-making Self-efficacy and Job Search Clarity and Moderation by Proactive Personality.*

**Author Response:** We sincerely thank the reviewer for this valuable and constructive feedback regarding the structure and completeness of the abstract. In response, we have revised the abstract to provide a clearer context, specify the study design, and enhance overall coherence. The following changes have been made:

Background: We added an opening sentence to highlight the significance of the topic and situate the study within the broader research context.

Added sentence: “*Career decision-making difficulties are a common challenge for college students, which can hinder their transition from education to employment. Based on the Social Cognitive Model of Career Self-Management, …*”

Methods: We clarified the research design to indicate that this study employed a cross-sectional quantitative approach.

**Revised sentence:** *“Based on the Social Cognitive Model of Career Self-Management, this study explored how social support was related to career decision-making difficulties. A total of 991 vocational college students participated in this cross-sectional quantitative study.”*

3. Reviewer’s comment: **Introduction**

*- **Background/Rationale**:*

*- Discusses Social Cognitive Model of Career Self-Management, Social Support and Career Decision-Making Difficulties, The Mediating Role of Career Decision-Making Self-Efficacy, The Mediating Role of Job Search Clarity, The Moderating Effect of Proactive Personality.*

*- **Objectives**:*

*- Clearly states the specific aims and the study hypotheses, but it is preferred to list all hypotheses one after the other not in between the text.*

**Author Response:** We appreciate the reviewer’s insightful comment and have revised the Introduction section accordingly to enhance clarity and logical flow. Following the reviewer’s suggestion, all hypotheses are now clearly listed together at the end of the Introduction. This improves readability and ensures that readers can easily grasp the overall research framework and logical relationships.

**Revised sentence:** …*The theoretical model of the study is shown in Figure 1 and hypotheses are as follows:*

*Hypothesis 1: Social support is negatively related to CDMD among vocational college students.*

*Hypothesis 2a: CDSE mediates the relationship between social support and vocational college students' CDMD.*

*Hypothesis 2b: Job search clarity plays a mediating role in the relationship between social support and CDMD among vocational college students.*

*Hypothesis 2c: CDSE and job search clarity play a serial mediating role in the relationship between social support and vocational college students’ CDMD.*

*Hypothesis 3a: Proactive personality positively regulates (moderated mediation ) the relationship between CDSE and CDMD. That is, the relationship between CDSE and CDMD is stronger when the level of proactive personality among vocational college students is higher.*

*Hypothesis 3b: Proactive personality positively regulates the relationship between job-search clarity and CDMD. That is, the relationship between job-search clarity and CDMD is stronger when the level of proactive personality among vocational college students is higher.*

4. Reviewer’s comment: **Methods**

- **Study Design**:

- Utilizes a cross-sectional survey was conducted, which is appropriate for examining variables.

- **Setting and Participants**:

- The study is conducted at a general hospital, with a clear description of the participant selection process.

- Sample size determination is not justified, moreover, what about the selection process and technique of participants.

- **Data Collection**:

- need more details about the year of development of each tool and revised versions

**Author Response:** We have revised the Methods section to clarify the cross-sectional study design, participant selection (convenience sampling), eligibility criteria, and rationale for sample size based on SEM guidelines. We also added information on the years of development and revisions for all measurement instruments. These modifications improve transparency, reproducibility, and methodological rigor.

5. Reviewer’s comment: **Results**

*- **Descriptive Statistics**:*

*- Presents demographic data of participants, including age, gender.*

*- **Correlation Analysis**:*

*- Provides clear statistical findings, highlighting significant correlations study variables*

**Author Response:** We have revised the Results section to explicitly present participant demographics and descriptive statistics. Tables now summarize gender, grade, means, standard deviations, and significant correlations among variables. These revisions improve clarity and align with your recommendations.

**Revised sentence:** *The sample consisted of 651 male students (65.69%) and 340 female students (34.31%), with 569 freshmen (57.42%), 328 sophomores (33.09%), and 94 juniors (9.49%). The correlation analysis results, presented in Table 2, …*

6. Reviewer’s comment: **Discussion**

*- **Key Findings**:*

*- Summarizes the main results in relation to the study objectives and hypotheses*

*- **Comparison with Existing Literature**:*

*- Compares findings with previous studies, noting both consistencies and novel contributions to the field.*

**Author Response:** We have revised the Discussion section to clearly summarize the key findings, explicitly linking them to the study objectives and hypotheses.

7. Reviewer’s comment: **Conclusion**

*- Reiterates the importance of enhancing social support, fostering job search clarity, and strengthening proactive personality traits to alleviate CDMD among vocational college students.*

**Author Response:** We have revised the Conclusion to explicitly emphasize the practical importance of enhancing social support, fostering job search clarity, and strengthening proactive personality traits.

8. Reviewer’s comment: **References**

*- The manuscript includes a comprehensive list of references, demonstrating engagement with current literature and foundational theories relevant to the study.*

**Author Response:** We appreciate the reviewer’s positive feedback regarding our references. We have ensured that the manuscript includes a comprehensive and up-to-date list of references, reflecting both foundational theories and recent empirical studies relevant to career decision-making, social support, self-efficacy, job search clarity, and proactive personality. This demonstrates our engagement with the current literature and supports the theoretical and empirical grounding of our study.

9. Reviewer’s comment: **Summary**

*The manuscript is well-structured and adheres to scientific research standards. It effectively addresses a pertinent issue among vocational college students.*

**Author Response:** We sincerely appreciate the reviewer’s positive evaluation of the manuscript’s structure and scientific rigor. We are glad that the study’s focus on career decision-making difficulties among vocational college students is recognized as both pertinent and relevant. Your acknowledgment reinforces the clarity and coherence of our presentation and encourages us to maintain high standards in conveying our research findings.

10. Reviewer’s comment: **To be added in limitation**

*But there are some mistake according scientific research steps: -**Data Collection***

*- **Reliance on Self-Reports**: Using subjective measures may lead to bias in responses*

***Sample***

*- **Sample Diversity**: If the sample consists only of vocational college students*

*, the results may not accurately reflect the experiences of all students across different vocational college*

Author Response: We sincerely thank the reviewer for highlighting the need to address methodological considerations related to data collection and sample diversity. We have expanded the limitations section to explicitly acknowledge the potential biases arising from reliance on self-report measures and the limited diversity of our sample consisting solely of Chinese vocational college students. Furthermore, we have emphasized the need for longitudinal designs, multi-informant data collection, and broader sampling strategies in future research to enhance generalizability and strengthen the robustness of findings. These revisions ensure greater transparency regarding the study’s constraints and provide clearer guidance for subsequent investigations.
