# Supplementary material for: The impact of social support on career decision-making difficulties: The serial mediating roles of career decision-making self-efficacy and job search clarity, and moderation by proactive personality
Source: PLoS One. 2026 Mar 9;21(3):e0344515. doi: 10.1371/journal.pone.0344515 (PMC12970892; doi:10.1371/journal.pone.0344515)
Supplement: Attachment — Submitted filename: Response_to_Reviewers_auresp_3.docx [file pone.0344515.s006.docx]

**Reviewer #3:**

1.Reviewer’s comment: ***Abstract***

**(1) Comment**: *The sentence: "Job search clarity partially mediate the relationship..." should be grammatically corrected to "partially mediates" to agree with the singular subject "Job search clarity".*

**Author Response**: Thank you for pointing out this grammatical error. We have corrected the verb form from "mediate" to "mediates" to ensure subject-verb agreement with the singular subject "Job search clarity". (See revised Abstract, Lines 18)

**(2) Comment**: *The phrase "The serial indirect effect was significant, β = -0.12, p < 0.001, accounting for 30% of the total effect." The negative beta indicates an inverse relationship, but clarity on the direction should be maintained.*

**Author Response**: Thank you for this crucial observation. You are absolutely correct that the negative beta coefficient requires clearer contextualization to avoid ambiguity. We have revised this sentence to explicitly state the directional meaning of the inverse relationship in the context of our serial mediation model.

The modified sentence now reads: "*The serial indirect effect was significant, β = -0.12, p < 0.001, accounting for 30% of the total effect, indicating that higher social support sequentially enhances career decision-making self-efficacy and job search clarity, which in turn reduces career decision-making difficulties.*" (See revised Abstract, Lines 22-24)

**(3) Comment**: *The abstract states, "they highlight the importance of designing career interventions for vocational college students that simultaneously target social support networks, self-efficacy enhancement, and clarity-building activities," which is clear but could benefit from specifying the practical implications more explicitly.*

**Author Response**: Thank you for this valuable suggestion to strengthen the practical significance of our findings. We have substantially expanded and specified the practical implications statement to provide clearer guidance for intervention development.

The revised sentence now reads: "*Practically, they highlight the importance of designing career interventions for vocational college students that simultaneously strengthen social support networks, enhance self-efficacy, and build job search clarity, with particular attention to students with lower proactive personality who may benefit most from such clarity-building activities.*"

2.Reviewer’s comment: ***Introduction***

***(1)* *Comment****. Missing or unclear hypotheses:*

*The hypotheses are now listed at the end, which improves clarity. However:*

*Hypothesis 2b: "Job search clarity plays a mediating role..." — The term "mediating role" should be consistent with the other hypotheses, and the structure should be parallel.*

*Hypotheses 3a and 3b: The wording "Proactive personality positively regulates (moderated mediation )..." contains an extra space before the parenthesis and the phrase "moderated mediation" should be hyphenated or clarified as "moderated mediation effect."*

**Author Response**: We appreciate this important observation regarding parallel structure and terminological consistency. We have revised Hypothesis 2b to align perfectly with the concise phrasing used in Hypotheses 2a and 2c.

We have made the following comprehensive revisions to Hypotheses 3a and 3b:

a) Removed extra spacing: Eliminated the redundant space before the parenthesis.

b) Clarified terminology: Replaced the ambiguous "positively regulates (moderated mediation)" with the precise, academically appropriate phrase "moderates the mediating role of". This clearly indicates a moderated mediation effect without needing parentheses or hyphens, aligning with contemporary statistical reporting standards.

c) Enhanced specificity: Added clarifying sentences to both hypotheses to explicitly state the nature of the moderation effect.

**Revised wording:**

***Revised Hypothesis 2b****:* "*Job search clarity mediates the relationship between social support and CDMD among vocational college students.*" (See revised Abstract, Lines 189)

**Revised Hypothesis 3a**: "*Proactive personality moderates the mediating role of CDSE in the relationship between social support and CDMD among vocational college students. Specifically, the indirect effect via CDSE is stronger when proactive personality is higher.*"

**Revised Hypothesis 3b**: "*Proactive personality moderates the mediating role of job search clarity in the relationship between social support and CDMD among vocational college students. Specifically, the mediating effect is stronger when proactive personality is higher.*"

***(2) Comment****. Clarity and consistency:*

*• The introduction mentions "The theoretical model of the study is shown in Figure 1." but the figure is not provided here; ensure it is clear in the final version.*

*• The hypotheses are somewhat complex; clearer language and consistent terminology (e.g., "moderated mediation" vs. "moderating role") would improve readability.*

**Author Response**: In the revised manuscript, we have explicitly inserted Figure 1 immediately following the hypotheses section in the Introduction.

We completely agree that hypothesis clarity is paramount. To address this, we have implemented a comprehensive terminology standardization and structural parallelization across all hypotheses.

3.Reviewer’s comment: **Methods**

***(1) Comment.*** *Study Design:*

*• The description states, "Utilizes a cross-sectional survey was conducted," which is ungrammatical. Correct phrasing would be: "A cross-sectional survey was conducted."*

**Author Response**: Thank you for identifying this grammatical error. We have standardized the opening sentence of Section 2.1 to read: "*A cross-sectional survey was conducted to examine...*" This eliminates any ungrammatical constructions and provides a clear, professional description of our study design throughout the manuscript. (See revised Methods, Lines 206)

***(2) Comment.*** *Participants:*

*• The description mentions "the study is conducted at a general hospital," which is inconsistent with the focus on vocational college students. This appears to be an error or a copy-paste mistake, likely from another study context.*

**Author Response:** We sincerely apologize for this significant error that somehow appeared in our manuscript. The mention of "general hospital" was indeed an inadvertent and inaccurate copy-paste mistake. We have corrected this to accurately reflect our actual research setting: "*The study was conducted at a vocational university in Chengdu, China*" (not a hospital). This correction ensures complete consistency with our study's focus on vocational college students. We have thoroughly checked the manuscript to eliminate any similar errors. (See revised Methods, Lines 208-210)

*• The sampling method is described as "convenience sampling," but the criteria for participant selection, inclusion/exclusion criteria, and the process are not detailed.*

**Author Response:** Thank you for this important suggestion to enhance methodological transparency. We have substantially expanded our participant description to provide comprehensive details:

Enhanced sampling clarity: We retained "convenience sampling" as an accurate description of our recruitment approach but added context that all enrolled students were invited to maximize sample diversity and generalizability.

Added detailed inclusion/exclusion criteria: We now explicitly specify: "*Participants were eligible if they met the following criteria: (a) full-time vocational college students, (b) currently enrolled in academic programs, (c) willing to provide informed consent, and (d) aged 18 years or older. Exclusion criteria included part-time students and those who had already secured full-time employment upon graduation.*" (See revised Methods, Lines 211-215)

*• The sample size of 991 is mentioned, but justification or power analysis to support this size is missing.*

**Author Response**: We appreciate this critical methodological point. We have added a rigorous sample size justification:

Referenced established SEM guidelines: We cited the "10–20 participants per estimated parameter" rule as our initial guideline. Added a priori power analysis: We conducted and reported Monte Carlo simulation results indicating that a sample size of 800 would provide adequate statistical power (≥ .80) to detect medium-sized mediation and moderation effects.

Explained oversampling rationale: We clarified that we aimed for 1000 participants to account for potential missing data and ensure robust parameter estimation, which resulted in our final sample of 991 (effective response rate = 84.56%).

This demonstrates that our sample size was determined through established statistical principles rather than convenience, addressing concerns about statistical power and generalizability. (See revised Methods, Lines 217-218)

***(3) Comment.*** *Data collection and instruments:*

*• The tools used (e.g., Perceived Social Support Scale, Career Decision-Making Self-Efficacy Questionnaire, etc.) are named, but:*

*o The development years or revision versions of these scales are not specified.*

*o Reliability and validity details of these instruments, especially within the current sample, are not provided.*

**Author Response**: We have comprehensively enhanced our measures section to provide complete psychometric transparency:

Specified original developers and revision years: For each scale, we now clearly state both the original authors/year and the specific Chinese revision scholars and years:

CDDQ: "The Chinese version of the Career Decision-making Difficulties Questionnaire (CDDQ, originally developed by Gati et al., 1996) [8], revised by Xue-Ping Shen in 2005 [41], was used for self-reporting."

PSSS: " The Perceived Social Support Scale(PSSS, originally developed by Zimet et al., 1988) [42], revised by Jiang Qianjin in 1999[43], was employed."

CDSE: " The "Career Decision-making Self-Efficacy Questionnaire for College Students (CDSE, originally developed by Taylor et al., 1983) [24]," developed by Y. X. Peng and L. R. Long in 2001[44], was used."

JSC: " The Job Search Clarity Scale (JSC), initially developed by Côté et al. (2006)[31] and revised by Wenhua Liao in 2007[45], was used. "

PPS: " The Proactive Personality Scale (PPS), originally developed by Bateman and Crant (1993)[36] and revised for the Chinese context by Chia-Yin Shang and Yi-Qun Gan in 2009[46], includes 11 items. "

Enhanced current sample reliability reporting: We standardized the reporting of internal consistency coefficients for the current sample using Cronbach's α, explicitly labeling them as "In the current sample..." to distinguish from original validation studies. For each scale, we provide both subscale and total scale reliabilities (e.g., CDDQ subscales α = 0.65, 0.96, 0.94; total α = 0.94).

*• The process of administering the questionnaires (e.g., online, paper-based), timing, and training of data collectors are not described.*

**Author Response**: We have expanded the data collection procedure description to include these essential details:

Administration method: Explicitly stated "all students enrolled in the university during the study period were invited to participate in a survey through an online platform." (See revised Methods, Lines 210-211)

Specific timing: Added " The study was conducted at a vocational university in Chengdu, China, from November 1 to November 30, 2023." (See revised Methods, Lines 209-210)

Data collector procedures: Clarified that "Data collection was coordinated with faculty members who were briefed on ethical protocols, though no formal training was required given the automated nature of the online survey"(See revised Methods, Lines 226-228)

***(4) Comment.*** *Statistical Analysis:*

*• The methods for data analysis (e.g., structural equation modeling, regression analyses, mediation/moderation testing) are not described in detail.*

**Author Response:** We have enhanced the statistical analysis section to provide greater methodological detail and clarity:

These enhancements ensure our analytical strategy is fully transparent and replicable. (See revised Methods, Lines 287-325)

*• No mention of software used (e.g., SPSS, AMOS, Mplus).*

**Author Response:** We have added a prominent statement at the beginning of the Statistical Analysis section: "*All statistical analyses were conducted using IBM SPSS 21.0 and Mplus 8.3.*" This critical information is now clearly stated rather than being embedded within the paragraph. (See revised Methods, Lines 276)

*• Handling of missing data or outliers is not addressed.*

**Author Response:** This is an important methodological detail that we have now comprehensively addressed:

Missing data and Outliers: Added "*Prior to hypothesis testing, data were screened for missing values, outliers, and multivariate normality. Missing data patterns were examined using Little's MCAR test. Given that missing data accounted for less than 5% of responses and were determined to be missing completely at random, listwise deletion was employed for primary analyses. Univariate outliers (values exceeding ±3.29 standard deviations) and multivariate outliers were identified and retained after confirming they represented legitimate extreme responses rather than data entry errors.* "

This demonstrates our rigorous approach to data quality assurance and addresses potential concerns about data integrity. (See revised Methods, Lines 276-282)

4.Reviewer’s comment: **Results**

*(1)* ***Comment****. Reporting of statistical findings:*

*• The beta coefficients (β) are reported, but confidence intervals are missing, which are essential for understanding the precision of estimates.*

**Author Response:** Thank you for this crucial suggestion. Although we had reported some confidence intervals in our original manuscript, we recognize that they were not systematically provided for all key findings. We have now added complete 95% bias-corrected bootstrap confidence intervals for every reported parameter estimate.

*• The effect sizes are mentioned as percentages (20%, 30%), but it's unclear how these were calculated or interpreted.*

**Author Response:** We apologize for this lack of clarity. We have now explicitly defined and explained the calculation of all relative mediation effects in both the text and Table 3. In Table 3, we added a footnote: "*Relative mediation effects were calculated as (indirect effect/total effect) × 100%.*"

In the narrative text, we have expanded the description to clearly state:. This transparent reporting allows readers to understand exactly how we quantified the magnitude of our mediated effects. (See revised Results, Section 3.3, and Table 3)

*• There is no mention of model fit indices or assumptions testing if structural equation modeling was used.*

**Author Response:** We appreciate this important methodological point. Although our original manuscript did include some model fit indices, we have now significantly expanded and systematized this reporting to ensure complete transparency:

Assumption testing added: We now explicitly report: "*Before conducting the mediation analysis, we assessed the assumptions underlying the mediation model. The Shapiro-Wilk test indicated approximately normal residuals (p > 0.05). Levene's test confirmed homogeneity of variance (p > 0.05).*" Additionally, we report post-hoc power analysis for non-significant findings.

Complete fit indices for all models: We now report comprehensive model fit indices for every SEM model tested, including:

Direct effect model: χ2 (18) = 56.24, RMSEA = 0.05, SRMR = 0.04, CFI = 0.99, TLI = 0.98

Mediation model: χ2 (112) = 375.93, RMSEA = 0.05, SRMR = 0.05, CFI = 0.98, TLI = 0.98

Moderated mediation models: χ2 (162) = 570.20, RMSEA = 0.05, SRMR = 0.08, CFI = 0.98, TLI = 0.97

Explicit interpretation: All fit indices are now explicitly interpreted as "good fitting" based on established criteria (CFI/TLI > 0.90, RMSEA/SRMR < 0.08).

This comprehensive reporting demonstrates that our models meet rigorous SEM standards. (See revised Results, Sections 3.3 and 3.4)

***(2) Comment.*** *Clarity and completeness:*

*• The results mention "the serial indirect effect was significant," but details on the mediation analysis (e.g., bootstrap confidence intervals) are absent.*

*• The moderation effect of proactive personality is described, but no figure or interaction plot is provided to illustrate the moderating effect.*

**Author Response:** We appreciate this suggestion for enhanced visualization. We would like to clarify that Figure 4 was indeed provided in our original submission, displaying the moderating effect of proactive personality on the relationship between job search clarity and CDMD. However, we recognize that its presence and interpretation could be more strongly emphasized.

Enhancements made:

Enhanced figure caption: The caption for Figure 4 now includes the simple slope values and confidence intervals, making it self-explanatory: "*Simple slope analysis shows: low proactive personality (β = -0.21, SE = 0.05, 95% CI [-0.32, -0.11]); high proactive personality (β = -0.54, SE = 0.08, 95% CI [-0.72, -0.41]).*"

Complementary textual description: The narrative now explicitly describes the pattern: "*The effect of job search clarity on reducing career decision-making difficulties became more pronounced as proactive personality increased.*"

We believe Figure 4, combined with the enhanced description, now provides a clear and comprehensive visual representation of our moderation findings. (See revised Results, Section 3.4, and Figure 4)

5.Reviewer’s comment: **Discussion and Conclusions**

***(1)comment****• The discussion section is not included in the excerpt, but potential errors to watch for in this section include:*

*o Overgeneralization of findings.*

**Author Response:** We have systematically reviewed our Discussion section and revised instances of overgeneralization by:

Adding precise statistical qualifiers to all findings (e.g., "negatively predicted CDMD" → "significantly and negatively predicted CDMD (β = -0.40, p < 0.001, 95% CI [-0.46, -0.34]), explaining 16.4% of the variance")

Qualifying our interpretations with appropriate caveats (e.g., changing "*this suggests that*" to "*this finding is consistent with the interpretation that*")

These changes ensure our conclusions are firmly anchored to our actual data rather than extending beyond what the evidence supports. (See revised Discussion throughout)

*o Lack of acknowledgment of limitations (e.g., cross-sectional design, sampling bias).*

**Author Response:** Thank you for this crucial observation. Our original limitations section, while present, was insufficiently detailed and lacked specific acknowledgment of key threats to validity. We have substantially expanded and strengthened the Limitations and Future Research Directions section to include. (See revised Discussion, Limitations subsection)

*o Speculative interpretations that are not supported by the data.*

**Author Response:** We have carefully reviewed our Discussion and removed or qualified all speculative statements by:

Adding citations to support any theoretical interpretations we propose

Providing alternative theoretical explanations when multiple interpretations are possible.

***(2)comment****. General Language and Formatting*

*• Several minor grammatical issues:*

*o Inconsistent capitalization (e.g., "Social Support" vs. "social support").*

**Author Response:** We have conducted a systematic review and standardization of capitalization throughout the entire manuscript. All variable names are now consistently presented in lowercase when used in general text (e.g., "social support," "career decision-making self-efficacy") and capitalized only when referring to specific scale names (e.g., "Perceived Social Support Scale," "Career Decision-Making Self-Efficacy Questionnaire") or at the beginning of sentences. This standardization eliminates the inconsistency you identified. (See revised manuscript throughout)

*o Punctuation inconsistencies, such as extra spaces before parentheses.*

**Author Response:** We have carefully scanned and corrected all punctuation errors, specifically:

Removed all extra spaces before parentheses throughout the manuscript.

Standardized spacing around mathematical operators and symbols

*• The transition sentences between sections are sometimes abrupt; ensure smooth flow.*

**Author Response:** We have enhanced transitional sentences at major section junctures to improve logical flow:

Between gender differences and social support sections: Added bridging sentence: "*This aligns with prior research indicating that gender can influence career decision-making processes and outcomes, highlighting the importance of considering gender-specific factors in vocational guidance interventions.*"

Between main effects and mediation sections: Added: "*These results suggest that social support operates through multiple pathways to influence career decision-making, and that enhancing social support systems may be a valuable strategy for improving vocational outcomes among students.*"

Between discussion and limitations: Added: " *Notwithstanding its substantive contributions to understanding CDMD among vocational college students...*"

These transitions create a more cohesive narrative arc and guide readers smoothly through our theoretical arguments. (See revised Discussion throughout)

**Reviewer #4:**

1. Reviewer’s comment: **Title**

The title clearly indicates the impact of Social Support on Career Decision- Making Difficulties: The Serial Mediating Role of Career Decision-making Self-efficacy and Job Search Clarity and Moderation by Proactive Personality.

**Author response:** We sincerely appreciate Reviewer’s positive assessment of our manuscript's title. We are pleased that the title effectively and clearly communicates the core focus of our study—the impact of social support on career decision-making difficulties, with the serial mediating roles of career decision-making self-efficacy and job search clarity, and the moderating role of proactive personality.

2. Reviewer’s comment: **Abstract**

- **Background**: acceptable context on the significance of the study

- **Objective**: Clearly states the aim of the study

- **Methods**: Describes the number of participants, including the study’s design

- **Results**: Summarizes key findings

- **Conclusion**: Highlights the impact of Social Support on Career Decision- Making Difficulties: The Serial Mediating Role of Career Decision-making Self-efficacy and Job Search Clarity and Moderation by Proactive Personality.

**Author response:** We thank Reviewer for their thorough review and positive assessment of our abstract. We are pleased that you found the abstract to be well-structured and effective in presenting.

3. Reviewer’s comment: **Introduction**

- **Background/Rationale**:

- Discusses Social Cognitive Model of Career Self-Management , Social Support and Career Decision-Making Difficulties , The Mediating Role of Career Decision-Making Self-Efficacy, The Mediating Role of Job Search Clarity, The Moderating Effect of Proactive Personality.

- **Objectives**:

- Clearly states the specific aims and the study hypotheses

**Author response:** We thank Reviewer for their positive assessment of our Introduction section. We are pleased that you found our Background/Rationale to comprehensively discuss the Social Cognitive Model of Career Self-Management and its key components (social support, career decision-making difficulties, career decision-making self-efficacy, job search clarity, and proactive personality), and that our Objectives clearly state the specific aims and study hypotheses.

4. Reviewer’s comment: **Methods**

- **Study Design**:

- Utilizes a cross-sectional survey was conducted, which is appropriate for examining variables.

- **Setting and Participants**:

- The study is conducted at a general hospital, with a clear description of the participant selection process.

**Author response:** We sincerely appreciate Reviewer's careful review of our Methods section. We are pleased that you found our study design appropriate for examining the variables and that our participant selection process was clearly described.

5. Reviewer’s comment: **Results**

- **Descriptive Statistics**:

- Presents demographic data of participants, including age, gender.

- **Correlation Analysis**:

- Provides clear statistical findings, highlighting significant correlations study variables

**Author response:** We thank Reviewer for their positive assessment of our Results section. We are pleased that you found our presentation of descriptive statistics (including demographic data on age and gender) and correlation analysis to be clear and effective in highlighting the significant relationships among study variables.

6. Reviewer’s comment: **Discussion**

- **Key Findings**:

- Summarizes the main results in relation to the study objectives and hypotheses

- **Comparison with Existing Literature**:

- Compares findings with previous studies, noting both consistencies and novel contributions to the field.

**Author response:** We sincerely appreciate Reviewer’s positive assessment of our Discussion section. We are pleased that you found it effectively summarizes the main results in relation to the study objectives and hypotheses, and that it appropriately compares our findings with previous studies, noting both consistencies and novel contributions to the field.

7. Reviewer’s comment: **Conclusion**

- Reiterates the importance of enhancing social support, fostering job search clarity, and strengthening proactive personality traits to alleviate CDMD among vocational college students

**Strengths and Limitations**

-this section to be considered

**Author response:** We thank Reviewer for their positive assessment of our Conclusion section. We are pleased that you found it effectively reiterates the importance of enhancing social support, fostering job search clarity, and strengthening proactive personality traits to alleviate CDMD among vocational college students.

Regarding the Strengths and Limitations section: We appreciate your suggestion to include this important component. In our revised manuscript, we have added a comprehensive "Limitations and Future Research Directions" subsection within the Discussion (Section 4.5). Additionally, we have incorporated a "Strengths of the Study" paragraph at the beginning of the Discussion to provide a balanced evaluation.

8. Reviewer’s comment: **References**

- The manuscript includes a comprehensive list of references, demonstrating engagement with current literature and foundational theories relevant to the study.

Summary

The manuscript is well-structured and adheres to scientific research standards. It effectively addresses a pertinent issue among vocational college students.

**Author response:** We are deeply grateful for Reviewer #4's comprehensive and positive evaluation of our manuscript. We particularly appreciate your recognition that our references demonstrate thorough engagement with current literature and foundational theories—a testament to our commitment to grounding this research in established scholarship while advancing new insights.

Thank you for your time, expertise, and supportive assessment of our work.
